# Prehospital vital sign monitoring in paediatric patients: an interregional study of educational interventions

**DOI:** 10.1186/s13049-023-01067-z

**Published:** 2023-01-14

**Authors:** Vibe Maria Laden Nielsen, Morten Breinholt Søvsø, Torben Anders Kløjgård, Regitze Gyldenholm Skals, Alasdair Ross Corfield, Lars Bender, Hans Morten Lossius, Søren Mikkelsen, Erika Frischknecht Christensen

**Affiliations:** 1 Centre for Prehospital and Emergency Research, Department of Clinical Medicine, Aalborg University Hospital, Aalborg University, Aalborg, Denmark; 2 Prehospital Emergency Services, Aalborg, North Denmark Region Denmark; 3 Unit of Clinical Biostatistics, Aalborg University Hospital, Aalborg, Denmark; 4 National Health Service Greater Glasgow and Clyde, University of Glasgow, Glasgow, UK; 5 Paediatric Department, Aalborg University Hospital, Aalborg, Denmark; 6 Norwegian Air Ambulance Foundation, University of Stavanger, Stavanger, Norway; 7 The Prehospital Research Unit, Odense University Hospital, Odense, Region of Southern Denmark Denmark

**Keywords:** Paediatric emergency medicine, Vital signs, Emergency medical services, Education and training, Triage, Clinical assessment, Paediatric readiness

## Abstract

**Background:**

Prehospital vital sign documentation in paediatric patients is incomplete, especially in patients ≤ 2 years. The aim of the study was to increase vital sign registration in paediatric patients through specific educational initiatives.

**Methods:**

Prospective quasi-experimental study with interrupted time-series design in the North Denmark and South Denmark regions. The study consecutively included all children aged < 18 years attended by the emergency medical service (EMS) from 1 July 2019 to 31 December 2021. Specific educational initiatives were conducted only in the North Denmark EMS and included video learning and classroom training based on the European Paediatric Advanced Life Support principles. The primary outcome was the proportion of patients who had their respiratory rate, peripheral capillary oxygen saturation, heart rate *and* level of consciousness recorded at least twice. We used a binomial regression model stratified by age groups to compare proportions of the primary outcome in the pre- and post-intervention periods in each region.

**Results:**

In North Denmark, 7551 patients were included, while 15,585 patients from South Denmark were used as a reference. Virtually all of the North Denmark EMS providers completed the video learning (98.7%). The total study population involved patients aged ≤ 2 months (5.5%), 3–11 months (7.4%), 1–2 years (18.8%), 3–7 years (16.2%) and ≥ 8 years (52.1%). In the intervention region, the primary outcome increased from the pre- to the post-intervention period from 35.3% to 40.5% [95% CI for difference 3.0;7.4]. There were large variations in between age groups with increases from 18.8% to 27.4% [95% CI for difference 5.3;12.0] among patients aged ≤ 2 years, from 33.5% to 43.7% [95% CI for difference 4.9;15.5] among patients aged 3–7 years and an insignificant increase among patients aged ≥ 8 years (from 46.4% to 47.9% [95% CI for difference − 1.7;4.7]). In the region without the specific educational interventions, proportions were steady for all age groups throughout the entire study period.

**Conclusions:**

Mandatory educational initiatives for EMS providers were associated with an increase in the extent of vital sign registration in paediatric patients ≤ 7 years. Incomplete vital registration was associated with, but not limited to non-urgent cases.

**Supplementary Information:**

The online version contains supplementary material available at 10.1186/s13049-023-01067-z.

## Introduction

Every tenth patient cared for by the Danish emergency medical services (EMS) is a paediatric patient, most often teenagers or infants and toddlers aged two years or younger [1, 2]. Life-threatening events are infrequent among children [3–6]. Nonetheless, clinical examination of children with acute illnesses or injuries can be challenging for health care professionals who do not treat children on a daily basis and may be stressful in emergency settings [7–10]. The Children's Safety Initiative-EMS identified several educational priorities for EMS providers: paediatric airway management, responder anxiety when working with children and general paediatric skills, among others [11]. There is an international trend of increasing ‘paediatric readiness’ among non-paediatric health care professionals in emergency settings, as this may reduce mortality from critical illness [12–14]. A paediatric triage model is embedded in the national electronic patient medical records (ePMR) system used by EMS providers in all of Denmark. Triage models, track-and-trigger systems and early warning scores are primarily based on vital signs. However, scores are often not complete in paediatric patients in EMS settings [15–18]. In the North Denmark EMS, nearly half of the children did not have one full set of vital signs documented in their prehospital ePMR [17]. One full set of vital signs was defined as all of the contemporaneous respiratory rate, peripheral capillary oxygen saturation (SpO_2_), heart rate and level of consciousness. This project had the overall goal of improving EMS providers’ abilities to examine and assess children via a number of educational interventions, including teaching a standardised approach to the child with acute illness and standardised advice for the administration of commonly used medications. The study objective was to evaluate if the educational initiatives were associated with an increase in the proportion of paediatric patients who had at least two full sets of vital signs obtained by EMS providers prior to arrival at a hospital. The initiatives focused primarily on clinical assessment of children aged less than two years, as vital sign registration is particularly deficient in this age group [15–17]. A secondary study objective was to investigate if patient factors or specific situations were associated with complete vital sign registration.

## Methods

### Study design and setting

This was a prospective interregional quasi-experimental study with an interrupted time-series design [19]. We assessed the primary outcome each month and compared the time periods before and after the implementation of the educational interventions in the North Denmark EMS. Any increase in the primary outcome could have been the result of a temporal trend. For this reason, we decided to compare the findings to those of the South Denmark EMS which then served as a reference within the same country. In the South Denmark EMS, no specific educational initiatives were conducted during the study period. The reporting of the study follows the ‘Strengthening the Reporting of Observational Studies in Epidemiology’ guidelines [20]. Patients were included consecutively during the study period 1 July 2019 to 31 December 2021. We used monthly aggregated data and split the total study period into 15 months before the educational initiatives commenced, the ‘pre-intervention period’, from 1 July 2019 to 30 September 2020; and 15 months after the educational initiatives commenced, the ‘post-intervention period’, from 1 October 2020 to 31 December 2021. The first 14 days of the post-intervention period were considered a start-up period and not included in the analyses.

The responsibility of health care in Denmark lies within the five health regions. Each region is responsible for operating publicly funded health care services including primary health care, hospitals and EMS. Accordingly, there are five regional Emergency Medical Coordination Centres (EMCCs) which command all prehospital units [21]. The health care professionals at the EMCC dispatch EMS resources according to the level of urgency, A to E, using a criteria-based decision support tool, the Danish Index for Emergency Care [22, 23]. Emergency calls are forwarded from the police to the EMCC via the national emergency number, 1–1-2, or from general practitioners or other health care professionals requiring the EMS. North Denmark and South Denmark EMS both cover mixed urban–rural areas. Although the South Denmark Region is larger than the North Denmark Region both geographically and in terms of population, the regions have a similar demographic structure where children aged < 18 years make up 19,7% (240,577/1,222,967) and 19,1% (112,514/589,837) of the inhabitants, respectively (2020Q3) [24]. The North and South Denmark Regions have similar EMS systems with ambulances staffed by two emergency medical technicians (EMTs) and rendez-vous mobile emergency care units with a paramedic and a prehospital physician specialised in anaesthesia and intensive care [21]. Generally, a physician-staffed mobile emergency care unit is engaged in about 20–25% of ambulance dispatches in both regions. The EMS systems operate within the same legal framework and provide the same standard care. All prehospital ground-level units use the same ePMR system nationally, and the system is integrated into the hospitals’ ePMR systems. In both regions, EMTs have been trained in paediatric advanced life support (ALS) before recruitment into the services. On top of their basic training, the educational efforts that existed before the study commenced differed for EMTs (350 providers) and paramedics (40–50 providers) in the intervention region. Before the interventions, mandatory training of paediatric ALS and discussion of paediatric case scenarios were scheduled for EMTs regularly. Paramedics have the competencies to administer certain pharmacological treatments, some independently and some delegated at the discretion of a prehospital physician. All providers were allowed duty-free time for completing any of the European Resuscitation Council’s courses, however, this was on his or her own incentive. Likewise for any re-certification. Hence, the study interventions would offer a systematic structure for some while being a mere brush-up for others. It was also compulsory for newly recruited providers. For prehospital physicians, e-learning was optional. From now on, the three professions are collectively designated ‘EMS providers’.

### Participants

Patients were included if they were aged < 18 years on the date when an ambulance was dispatched from the EMCC, either requested via 1–1-2 or by a general practitioner or another health care professional. Exclusion criteria were calls classified as urgency level D, ‘patient needed transport in a supine position, no treatment needs’, or level E, ‘other mode of transport, e.g. taxi, or guidance to other acute care options’; interfacility transfers; duration of mission was less than 30 s; no patient was found at the scene; helicopter EMS was the first unit at the scene (as they had a different ePMR system); the patient was declared dead at the scene according to the prehospital ePMR or the patient had received basic life support. All of the above exclusion criteria were selected based on the assumption that EMS providers would not perform a formal ABCDE evaluation (including the recording of vital signs) of the patients in those scenarios.

### Intervention

The first initiative launched was an instructional video demonstrating the ABCDE approach by use of a 1-year-old manikin based on the European Resuscitation Council’s Guidelines on paediatric advanced life support principles (2015 edition [25] updated by the 2021 edition [26] with written consent from the council). The video instructed EMS providers to obtain two sets of vital signs to detect any changes: one during the primary assessment of the patient and one shortly before arrival at hospital. A full set of vital signs included respiratory rate, SpO_2_, heart rate and level of consciousness. The video was released and distributed as an e-learning session on 21 September 2020, with instructions to have completed the session by 14 October 2020. The cut-off date dividing the two study periods was 1 October 2020. Halfway through the post-intervention period, reminder lessons were held at the annual mandatory educational sessions. In collaboration with representatives of the EMS providers, we provided written calculation rules and standardised recommendations for administration of commonly used medications in paediatric emergency medicine (electronic and printed versions). The recommendations were uploaded to the application platform that the EMS providers are able to consult in their daily practice. The final version was reviewed and approved by a consultant in paediatrics (LB), a consultant in paediatric anaesthesiology (SK), the medical director of the North Denmark EMS (MR-K) and the daily manager of the physician-staffed mobile emergency care unit (MD). Please refer to the Acknowledgement section for details.

### Data collection and outcomes

Data were included exclusively from databases. Time stamps and locations of dispatched prehospital units were collected from the logistic system, Logis CAD (*Logis Solutions, Nærum, Denmark*), while patient data were collected from the prehospital ePMR system. Data collectors were all EMS providers employed in the two regions’ EMS. The Danish Regions’ Paediatric Triage Model (displayed in Additional file 1) and the Danish Emergency Process Triage [27] are integrated into the national ePMR system. Ranges of vital signs for paediatric patients are identical in the two triage systems, and the triage score is represented by colours: green for ‘not urgent’, yellow for ‘less urgent’, orange for ‘urgent’ and red for ‘life-threatening’ emergencies. The North Denmark EMS replaced their LIFEPAK® 15 monitors (©*Physio-Control, Inc., WA 98052, USA*) with ZOLL® X Series® monitors (*ZOLL Medical Corporation, MA 01824, USA*) on 1 December 2020. The new monitors automatically transferred values of SpO_2_ and heart rate into the prehospital ePMR during continuous monitoring whereas the former monitor required manual activation for the transfer of data into the record. For both monitors, respiratory rate required manual entry into the ePMR, except for when measured by capnography. Heart rate could be transferred into the ePMR either from a rhythm monitor or from a pulse oximeter. The primary outcome was registration of *two sets* of the following vital signs: respiratory rate, SpO_2_, heart rate *and* level of consciousness, hereafter designated ‘complete vital sign registration’. Level of consciousness was registered using either the Glasgow Coma Scale or the AVPU scale (Alert–Voice–Pain–Unresponsive) [28]. We incorporated a paediatric Glasgow Coma Scale into the prehospital ePMR system with separate verbal responses in children aged 5 years or younger and children aged 6 or above.

### Study size

The sample size was calculated using a formula for comparing proportions in two independent study populations with a dichotomous primary endpoint. Calculations were based on the following conditions: 1) the incidence of the primary outcome ‘two full sets of vital signs’ had previously been 36% (historical data from the prehospital ePMR database); 2) we aimed for a minimum of 10% increase relative to the pre-intervention period and 3) alpha = 0.05 and (1 − beta) = 0.80. This calculation provided a sample size of 5652 patients from the intervention region.

### Analysis

Data were pseudo-anonymised before analysis. Accidents were defined using dispatch criteria according to the Danish Index for Emergency Care [22, 23]. Chapters ‘04: Large scale accident’, ‘32: Traffic accident’ and ‘33: Accidents (not traffic-related)’ were collectively designated ‘Trauma’ while chapter ‘31: Minor wounds—fractures—injuries’ was designated ‘Minor injuries’. The primary outcome of having two full sets of vital signs registered is presented graphically as proportions according to pre- and post-intervention periods and per region. The graphic illustration is supported by a binomial regression model with robust variance estimation comparing the proportion of the primary outcome in the pre- and post-intervention periods. The model was stratified by age groups according to the Danish Regions’ Paediatric Triage Model displayed in Additional file 1 and reported by relative and absolute differences with 95% confidence intervals written as [xx;xx]. Sensitivity analyses of the primary outcome were performed with first-time events only. We used DAGitty [29] to illustrate directed acyclic graphs for discussion of possible confounders for the primary outcome. We agreed to adjust for Coronavirus disease of 2019 (COVID-19) incidence peaks, as the post-intervention period contained more months in the winter season than the pre-intervention period. Peaks were defined as months with a national lockdown of schools more than 50% of the time due to the risk of COVID-19 infection. We also adjusted for treat-and-release situations as vital signs may not be recorded more than once in such situations. And the proportion of treat-and-release situations may have differed between regions. We investigated if the following factors were associated with complete vital sign registration: age group, sex, EMS care time, treat-and-release situations, COVID-19 incidence peaks, implementation of new monitors, Glasgow Coma Score, triage score, prehospital physician present at the scene, accidents and critical interventions. Factor associations are presented by relative risks and risk differences compared to the relevant reference group for each factor. Data analyses were performed with Stata/MP 17.0 (*StataCorp LLC, TX 77845, USA*).

## Results

### Characteristics of study subjects

**Fig. 1:**
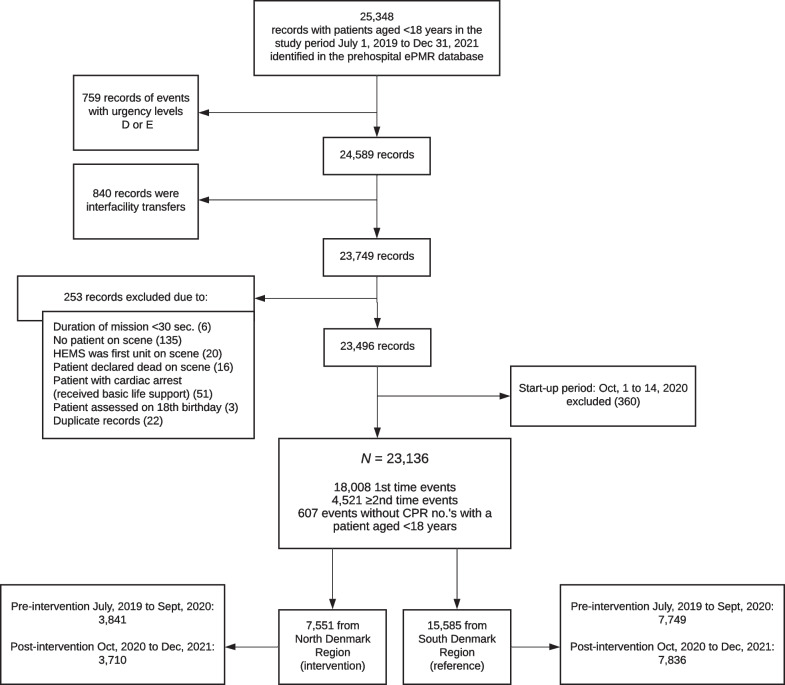
Patient flow diagram. *ePMR* electronic patient medical record. *HEMS* helicopter emergency medical services. *CPR* Civil Personal Registry

In the first 10 days following the launch of the initiatives, 81.8% of the service’s EMS providers had completed the e-learning session. This proportion had increased to 98.7% by the end of the study period. The patient flow is depicted in Fig. 1. The final study population included 7551 patients from the intervention region (North Denmark) and 15,585 patients from the reference region (South Denmark). The two regions had similar annual incidences of calls to the EMCC regarding paediatric patients (per 1000 inhabitants < 18 years) (Table 1). Overall, a prehospital physician had been dispatched along with the ambulance in 38.4% of cases. There was no difference in demographic variables such as sex and age groups. A third of the patients were infants and toddlers aged ≤ 2 years and half of the patients were aged 8 years or above. First triage scores varied between regions with more patients in the ‘urgent’ categories in the intervention region compared to the reference region (Table 1).

**Table 1:** Baseline characteristics by region

	North Denmark EMS (intervention)		South Denmark EMS (reference)	
	(n = 7551)		(n = 15,585)	
*Annual regional incidence of emergency calls regarding children*				
Calls per 1000 inhabitants < 18 years	30		29	
*Sex, n (%)*				
Male	3957	(52.4)	8144	(52.3)
Missing data, n (%)	121	(1.6)	204	(1.3)
*Age, n (%)*				
0–2 months	419	(5.5)	850	(5.5)
3–11 months	583	(7.7)	1138	(7.3)
1–2 years	1463	(19.4)	2882	(18.5)
3–7 years	1278	(16.9)	2459	(15.8)
8–17 years	3808	(50.4)	8256	(53.0)
Missing data, n (%)	0	(0.0)	0	(0.0)
*Response time*^a^				
Median (IQR), minutes	11	(7–19)	7	(4–11)
Missing data, n (%)	603	(8.0)	880	(5.6)
*Treat-and-release situations, n (%)*				
Against EMS provider’s advice	200	(2.6)	249	(1.6)
EMS provider’s initiative	975	(12.9)	1811	(11.6)
Missing data, n (%)	0	(0.0)	0	(0.0)
*EMS care time*^b^, n (%)				
0–15 min	277	(4.3)	400	(3.0)
15–30 min	1378	(21.6)	2407	(17.8)
30–45 min	1522	(23.9)	4046	(29.9)
45–60 min	1262	(19.8)	2977	(22.0)
60–120 min	1117	(17.5)	1815	(13.4)
≥ 120 min	93	(1.5)	272	(2.0)
Missing data, n (%)	727	(11.4)	1608	(11.9)
*Accidents, n (%)*				
Trauma	1527	(20.2)	3416	(21.9)
Minor injuries	345	(4.6)	531	(3.4)
Missing data, n (%)	299	(4.0)	401	(2.6)
*First triage score*^c^, n (%)				
Life-threatening (red)	415	(5.5)	717	(4.6)
Urgent (orange)	1682	(22.3)	2328	(14.9)
Less urgent (yellow)	1456	(19.3)	2851	(18.3)
Not urgent (green)	2073	(27.5)	6266	(40.2)
Missing data, n (%)	1925	(25.5)	3423	(22.0)

*EMS* emergency medical services; *IQR* interquartile range

^a^Time from dispatch to arrival at scene

^b^Time from arrival at scene to arrival at hospital for patients that were not released at the scene (n = 6376 in the intervention region and n = 13,525 in the reference region)

^c^Triage score according to the Danish Regions’ Paediatric Triage Model, and if this field on the prehospital ePMR was empty, then according to the Danish Emergency Process Triage (DEPT)

### Main results

**Fig. 2:**
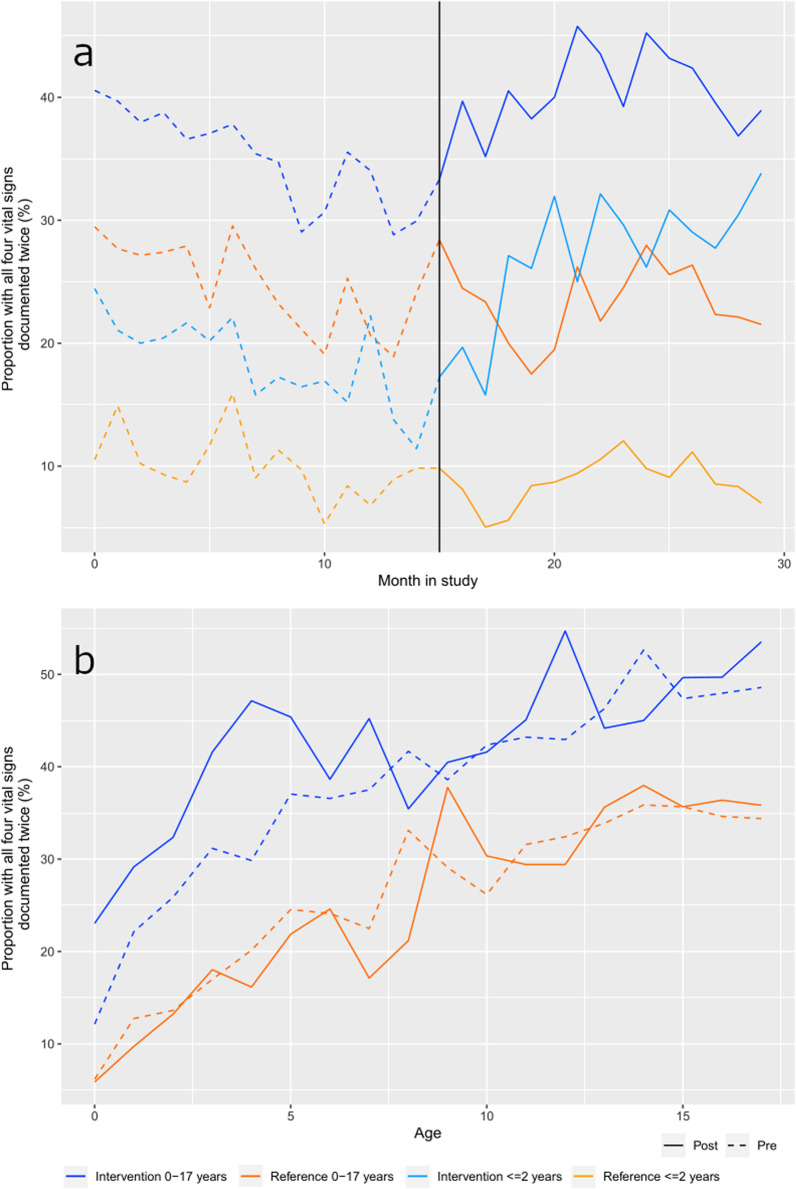
Primary outcome by time (**a**) and by age (**b**). The vertical line represents the cut-off date from the pre- to the post-intervention period. Full population: *N* = 23,136. Age ≤ 2 years: n = 7335

The vital signs of interest were respiratory rate, SpO_2_, heart rate and level of consciousness. The primary outcome of complete vital sign registration increased in the intervention region from the pre- to the post-intervention period and compared to the reference region (Fig. 2). There were large variations in between age groups (Table 2). For children of all ages, the proportion of patients with complete vital sign registration was 40.5% [38.9;42.0] in the intervention region, and 23.7% [22.7;24.6] in the reference region in the post-intervention period. Sensitivity analyses of the primary outcome with first-time events only (n = 18,008) produced similar unadjusted results. Adjustment for COVID-19 national lockdown periods and treat-and-release situations did not change our results notably (Table 2). In the intervention region, more patients experienced changes in vital signs and more patients improved their vital signs during the prehospital phase in the post-intervention period compared to the pre-intervention period (please refer to Additional file 2).

**Table 2:** Complete vital sign registration during the pre- versus post-intervention periods by region (*N* = 23,136)

	Proportions, %		Unadjusted results					Adjusted results^a^				
	Pre-	Post-	Relative risk [95% CI]		Absolute difference, %-points [95% CI]			Relative risk [95% CI]		Absolute difference, %-points [95% CI]		
	intervention											
*All ages (N = 23,136)*												
Post vs. pre-intervention period, intervention region	35.3	40.5	1.1	[1.1;1.2]	5.2	[3.0;7.4]	*p* < 0.01	1.2	[1.1;1.2]	5.4	[3.2;7.5]	*p* < 0.01
Post vs. pre-intervention period, reference region	24.7	23.7	1.0	[0.9;1.0]	− 1.1	[− 2.4;0.3]		1.0	[0.9;1.0]	− 0.9	[− 2.2;0.4]	
*0–2 months (n = 1269)*												
Post vs. pre-intervention period, intervention region	5.4	12.7	2.3	[1.2;4.5]	7.3	[1.8;12.8]	*p* = 0.01	2.2	[1.1;4.3]	7.5	[2.1;12.9]	*p* = 0.02
Post vs. pre-intervention period, reference region	3.3	2.8	0.9	[0.4;1.9]	− 0.4	[− 2.7;1.9]		0.9	[0.4;1.9]	− 0.4	[− 2.7;1.9]	
*3–11 months (n = 1721)*												
Post vs. pre-intervention period, intervention region	17.3	30.2	1.7	[1.3;2.4]	12.9	[6.1;19.8]	*p* < 0.01	1.8	[1.3;2.4]	13.5	[6.7;20.3]	*p* < 0.01
Post vs. pre-intervention period, reference region	8.6	8.0	0.9	[0.6;1.4]	− 0.6	[− 3.9;2.6]		0.9	[0.6;1.4]	− 0.6	[− 3.8;2.6]	
*1–2 years (n = 4345)*												
Post vs. pre-intervention period, intervention region	23.4	30.3	1.3	[1.1;1.5]	6.9	[2.4;11.4]	*p* < 0.01	1.3	[1.1;1.5]	7.0	[2.5;11.5]	*p* < 0.01
Post vs. pre-intervention period, reference region	13.0	11.1	0.9	[0.7;1.0]	− 1.9	[− 4.3;0.5]		0.9	[0.7;1.0]	− 1.8	[− 4.2;0.6]	
*3–7 years (n = 3737)*												
Post vs. pre-intervention period, intervention region	33.5	43.7	1.3	[1.1;1.5]	10.2	[4.9;15.5]	*p* < 0.01	1.3	[1.2;1.5]	10.9	[5.6;16.2]	*p* < 0.01
Post vs. pre-intervention period, reference region	20.9	19.1	0.9	[0.8;1.1]	− 1.8	[− 5.0;1.4]		0.9	[0.8;1.1]	− 1.5	[− 4.6;1.6]	
* ≥ 8 years (n = 12,064)*												
Post vs. pre-intervention period, intervention region	46.4	47.9	1.0	[1.0;1.1]	1.5	[− 1.7;4.7]	*p* = 0.69	1.0	[1.0;1.1]	1.6	[− 1.5;4.8]	*p* = 0.68
Post vs. pre-intervention period, reference region	33.7	34.5	1.0	[1.0;1.1]	0.7	[− 1.3;2.8]		1.0	[1.0;1.1]	0.8	[− 1.2;2.9]	

*p*-values refer to differences between regions. No missing data

^a^Adjusted for months with COVID-19 lockdown of schools > 50% of the time and treat-and-release situations

Age was markedly associated with the primary outcome. The youngest patients were less likely to have had complete vital sign registration (Table 3). EMS providers in the intervention region recorded at least one respiratory rate in 76.5% [74.1;78.8] of cases, heart rate in 71.5% [68.9;74.0] of cases, SpO_2_ in 68.9% [66.2;71,4] of cases and level of consciousness in 78.3% [75.8;80.5] of cases within the subgroup of patients aged ≤ 2 years during the post-intervention period. Treat-and-release situations were also associated with the primary outcome. Treat-and-release situations represented 21.4% [19.7;23.2] of the cases with incomplete registration in the intervention region and 15.4% [14.5;16.4] in the reference region. The introduction of new monitors in the intervention region during the study period was not associated with a change in the proportion of patients with complete vital sign registration. Having a ‘non-urgent’ (green) triage score at the time of the first assessment decreased the probability of complete vital sign registration compared to the patients with ‘urgent’ scores (Table 3). In the intervention region, the patients with incomplete vital sign registration were triaged as’not urgent’ (green) in 25.8% [23.9;27.6] of the cases and as ‘urgent’ (yellow, orange or red) in 36.8% [34.7;38.8] of the cases during the post-intervention period (missing triage score from the prehospital ePMR in 37.5% [35.5;39.5]).

**Table 3:** Factors associated with complete vital sign registration during EMS care time in the post-intervention period

	Intervention region (n = 3710)		Reference region (n = 7836)	
	Relative risk compared to ref. [95% CI]	Risk difference compared to ref. [95% CI]	Relative risk compared to ref. [95% CI]	Risk difference compared to ref. [95% CI]
*Age group*				
0–2 months	0.3 [0.2;0.4]	− 0.35 [− 0.40; − 0.30]	0.1 [0.0;0.1]	− 0.32 [− 0.34; − 0.29]
3–11 months	0.6 [0.5;0.8]	− 0.18 [− 0.23; − 0.12]	0.2 [0.2;0.3]	− 0.26 [− 0.29; − 0.24]
1–2 years	0.6 [0.6;0.7]	− 0.18 [− 0.22; − 0.14]	0.3 [0.3;0.4]	− 0.23 [− 0.25; − 0.21]
3–7 years	0.9 [0.8;1.0]	− 0.04 [− 0.09; 0.00]	0.6 [0.5;0.6]	− 0.15 [− 0.18; − 0.13]
8–17 years (= ref)	1	–	1	–
Missing data, n (%)	0 (0.0%)		0 (0.0%)	
*Sex*				
Female	1.0 [1.0;1.1]	0.01 [− 0.02; 0.04]	1.1 [1.0;1.1]	0.01 [− 0.01; 0.03]
Male (= ref)	1	–	1	–
Missing data, n (%)	35 (0.9%)		65 (0.8%)	
*EMS care time*^a^				
0–15 min	0.6 [0.4;0.8]	− 0.21 [− 0.29; − 0.13]	0.5 [0.4;0.7]	− 0.13 [− 0.18; − 0.08]
> 15 min (= ref)	1	–	1	–
Missing data, n (%)	370 (11.9%)		751 (11.1%)	
*Treat-and-release situations*				
Yes	0.5 [0.4;0.5]	− 0.24 [− 0.28; − 0.20]	0.6 [0.5;0.7]	− 0.11 [− 0.13; − 0.08]
No (= ref)	1	–	1	
Missing data, n (%)	0 (0.0%)		0 (0.0%)	
*COVID-19*				
Months with school lockdown in place > 50% of the time^b^	1.0 [0.9;1.1]	− 0.01 [− 0.06;0.03]	0.8 [0.7;0.9]	− 0.04 [− 0.07; − 0.02]
Months without lockdown (= ref)	1	–	1	–
Missing data, n (%)	0 (0.0%)		0 (0.0%)	
*Implementation of ZOLL® monitors during the post-intervention period*^c^				
2 months following implementation date	1.0 [0.8;1.2]	0.01 [− 0.06;0.08]	*NA*	*NA*
2 months before implementation date (= ref)	1	–		
Missing data, n (%)	0 (0.0%)			
*Glasgow Coma Score (first observation)*				
Green = normal LOC	1.0 [0.9;1.2]	0.02 [− 0.04; 0.08]	0.7 [0.7;0.8]	− 0.10 [− 0.14; − 0.05]
Yellow/orange/red = altered LOC (= ref)	1	–	1	–
Missing data, n (%)	571 (15.4%)		1513 (19.3%)	
*Triage score (first)*				
Green = ‘Not urgent’	0.8 [0.7;0.9]	− 0.11 [− 0.14; − 0.07]	0.8 [0.7;0.9]	− 0.07 [− 0.09; − 0.05]
Yellow/orange/red = ‘Less urgent’/’Urgent’/’Life-threatening’ (= ref)	1	–	1	–
Missing data, n (%)	925 (24.9%)		1758 (22.4%)	
*Prehospital physician at the scene*				
Yes, HEMS or MECU physician	0.8 [0.7;0.9]	− 0.08 [− 0.11; − 0.04]	0.7 [0.7;0.8]	− 0.08 [− 0.10; − 0.06]
No (= ref)	1	–	1	–
Missing data, n (%)	0 (0.0%)		0 (0.0%)	
*Accidents*^d^				
Trauma	1.2 [1.1;1.3]	0.06 [0.02;0.10]	1.1 [1.0;1.3]	0.03 [0.01;0.06]
Minor injuries	1.4 [1.2;1.6]	0.15 [0.07;0.23]	1.6 [1.4;1.9]	0.14 [0.08;0.20]
Not accident (= ref)	1	–	1	–
Missing data, n (%)	137 (3.7%)		136 (1.7%)	
*Critical intervention(s)*^e^				
≥ 1 intervention(s) performed	1.6 [1.4;1.7]	0.22 [0.17;0.27]	2.2 [2.0;2.4]	0.25 [0.22;0.28]
None (= ref)	1	–	1	–
Missing data, n (%)	0 (0.0%)		0 (0.0%)	

*COVID-19* Coronavirus disease of 2019*. EMS* emergency medical services. *HEMS* helicopter emergency medical services. *LOC* level of consciousness. *MECU* mobile emergency care unit. *NA* not applicable

^a^Time from arrival at scene to arrival at hospital for patients that were not released at the scene (n = 3117 in the intervention region and n = 6758 in the reference region)

^b^Months: January 2021, February 2021, December 2021

^c^The North Denmark EMS replaced their LIFEPAK® monitors with ZOLL® monitors on 1 December 2020 (refer to the ‘Data collection and outcomes’ paragraph)

^d^Accidents as defined in Table 1: Trauma defined as accidents within chapters ‘04: Large scale accident’ or ‘32: Traffic accident’ or ‘33: Accidents (not traffic-related)’. Minor injuries defined as accidents within chapter ‘31: Minor wounds—fractures—injuries’

^e^Critical interventions include peripheral venous catheter, intraosseous vascular access, intubation or bag-valve-mask ventilation

## Discussion

### Key results

Following the introduction of the educational initiatives, the proportion of patients with complete vital sign registration increased compared to the reference region though the increase clearly differed by age group. For children of all ages, a marked difference was observed between regions in the post-intervention period where 40.5% and 23.7% had complete vital sign registration in the intervention and reference region, respectively.

### Limitations

A causal relationship between the educational interventions and the outcome cannot be concluded with a quasi-experimental study design. We considered a randomised cluster design within our service using individual ambulance stations. However, this could be impractical as the EMS providers may have exchanged knowledge and possibly created a spill-over effect between groups. And when providers had been educated, it would not be possible to ‘unlearn’ the material. This suggested choosing a parallel design over a crossover design. We decided to divide the study groups both by period and by regional service level, and our study findings demonstrate improvements in both comparisons. The results were not biased by the replacement of monitors during the post-intervention period in the intervention region. It is possible that the EMS providers were inclined to change registration practices as a result of being studied and not because of the educational initiatives. The increase in the primary outcome persists all through the post-intervention period which contradicts a strong Hawthorne effect [30] in our study, as the post-intervention period lasted for more than a year. All events in our study were not strictly independent from each other as a unique patient could have had more than one ambulance dispatched during the entire study period. However, we suspected that this would not change the results, as it would probably not be the same EMS providers that would be dispatched to a unique patient multiple times. A call to the EMCC concerning a child, who had already had an ambulance dispatched during the study period, was regarded as either a new emergency or a progression of the situation. The sensitivity analyses support the abovementioned presumption.

We defined ‘complete vital sign registration’ as a minimum of two sets of the four vital signs respiratory rate, SpO_2_, heart rate and level of consciousness. This was an ambitious outcome compared to the existing literature. Vital signs were not always registered independently, as heart rate and SpO_2_ would often have been measured simultaneously using a pulse oximeter. Those four vital signs were chosen because of their association with clinical outcomes in paediatric patients in emergency settings [16, 31, 32] and because of their simplicity and mild discomfort for the child. Hypotension is a specific, yet late sign of circulatory failure. We did not expect blood pressure to be measured in all paediatric patients, as readings are dependent on appropriate cuff size and can be falsely raised in crying or agitated children [33–35]. If a vital sign was not registered in the prehospital ePMR’s designated vital sign entry field, we considered it not to have been measured or assessed. This may have caused us to underestimate the primary outcome. Integrating compulsory entry fields into the prehospital ePMR may be a way of increasing vital sign registration. This option had however been discussed previously by a national board at the time of implementation of the national prehospital ePMR. The board considered it would not be feasible to have the prehospital ePMR ‘locked’ on a previous mission with unfilled entry fields, should another mission have priority.

### Interpretation and generalisability

The study is strengthened by its population-based design including both patients transported to a hospital and treat-and-release patients. The consecutive inclusion of patients minimised selection bias. The generalisability of the results might be influenced by local practices and work cultures in different EMS though. The North Denmark EMS aimed to increase complete vital sign registration in children. But what is an acceptable level? In the post-intervention period, the primary outcome of two full sets of vital signs was 41% in our service with large variation between age groups. In a national cohort of more than 100,000 paediatric EMS patients in the United Kingdom, 62% had those same four vital signs documented in their prehospital ePMR at least once [16]. The authors advocate for a simpler paediatric early warning score in the prehospital setting as oxygen delivery, Glasgow Coma Score, heart rate and SpO_2_ were able to predict 30-day mortality or intensive care admission within 48 h as accurately as a full score that included measures of both respiratory rate, SpO_2_, oxygen delivery, temperature, systolic blood pressure, heart rate, time to capillary reperfusion and level of consciousness [4, 16].

The main findings revealed that the younger the patient, the lower the probability of complete vital sign registration, and similar age variations are well documented [15, 16, 18, 36, 37]. Infants are at the highest risk of experiencing non-accidental injuries [38] that must not go unnoticed by the EMS. Our findings reflect that the educational initiatives for this project were centred around the clinical examination of infants and small toddlers. The extent of individual vital sign registration within our service is acceptable for infants and toddlers if the results are compared to other prehospital systems in the Nordic countries [6, 18]: respiratory rate 34–51% (77% in our service), heart rate 59–66% (72% in our service), SpO_2_ 56–69% (69% in our service) and level of consciousness 29–83% (78% in our service). In United States EMS, registration rates of respiratory rate (81–89%) and heart rate (57–91%) appear to be higher [15, 36, 37]. However, these studies all report on registration of single vital signs separately, while this study reports on complete vital sign registration as a proxy for a basic ABCD evaluation and reevaluation of the patient. Patients with a ‘non-urgent’ triage score had a relative risk of 0.8 for complete vital sign registration compared to patients with ‘urgent’ scores. Yet, incomplete vital sign registration was not restricted to non-urgent cases. This supports the findings from a previous smaller sample [17]. Although we observed adequate increases within four of the five age groups, obviously there is still a potential for increasing vital sign registration in our service. However, increasing vital sign registration is not equal to improving outcomes. Choosing appropriate outcomes for clinical improvement is complicated, as ‘typical’ patient-centred outcomes in an adult population, such as ICU admittance or death, are much more infrequent in paediatric populations. As a surrogate outcome measure for change in clinical condition during the prehospital phase, we compared the first set of vital signs to the last set. More patients experienced changes, and more patients improved their vital signs in the post-intervention period compared to the pre-intervention period (Additional file 2). The study is well in accordance with the trend of increasing ‘paediatric readiness’ among non-paediatric health care professionals, and the educational initiatives may have had derivative effects. We provided written standardised recommendations for commonly used medications in paediatric emergency medicine, ‘which has been shown to reduce administration errors’ [18]. EMS providers were encouraged to have a standardised approach to the clinical examination of children, and this may have increased the caregivers’ confidence in the ambulance team [10].

## Conclusions

Our study implies that mandatory educational initiatives for EMS providers contribute to a more thorough examination of paediatric patients ≤ 7 years by increasing the extent of vital sign registration. Incomplete vital registration was associated with, but not limited to non-urgent cases. The findings indicate that Danish EMS could benefit from regular in-service training in paediatric emergencies in order to advance ‘paediatric readiness’.

## Supplementary Information

**Additional file 1**: The Danish Regions’ Paediatric Triage Model.

**Additional file 2**: Differences between triage scores calculated from the first and last set of vital signs in the intervention region (North Denmark Region).

## Data Availability

The data that support the findings of this study are available from the North Denmark Region but restrictions apply to the availability of these data, which were used under license for the current study, and so are not publicly available. Data are however available from the authors upon reasonable request and with permission of the North Denmark Region.

## Abbreviations

ABCDE: Airways–Breathing–Circulation–Disability–Exposure
ALS: Advanced life support
AVPU: Alert–Voice–Pain–Unresponsive
COVID-19: Coronavirus disease of 2019
EMCC: Emergency medical coordination centre
EMS: Emergency medical services
EMT: Emergency medical technician
ePMR: Electronic patient medical records
SpO_2_: Peripheral capillary oxygen saturation
