# Supplementary material for: Prehospital vital sign monitoring in paediatric patients: an interregional study of educational interventions
Source: Scand J Trauma Resusc Emerg Med. 2023 Jan 14;31:4. doi: 10.1186/s13049-023-01067-z (PMC9839956; doi:10.1186/s13049-023-01067-z)
Supplement: Supplementary file 1 — Additional file 1: The Danish Regions’ Paediatric Triage Model. [file 13049_2023_1067_MOESM1_ESM.pdf]

Additional file 1. The Danish Regions' Paediatric Triage Model used in the emergency medical services (Danish Regions, 2012)

Paediatric Triage Model

Triage level	1 Life-threatening	2 Urgent	3 Less urgent	4 Not urgent
A	Airway obstruction	Partial airway obstruction	Partial airway obstruction only at physical activity	No airway obstruction
B	SpO_2 < 85% without O_2<br>SpO_2 < 90% with O_2<br>Respiratory rate red	SpO_2 85-92% without O_2<br>SpO_2 90-96% with O_2<br>Respiratory rate orange	SpO_2 93-94% without O_2<br>SpO_2 ≥ 97% with O_2<br>Respiratory rate yellow	SpO_2 ≥ 95% without O_2<br>Respiratory rate green
C	Heart rate red<br>Capillary refill time > 4 sec*	Heart rate orange<br>Capillary refill time 4 sec*	Heart rate yellow<br>Capillary refill time 3 sec*	Heart rate green<br>Capillary refill time ≤ 2 sec*
D	Unconscious<br>GCS ≤ 8	Altered level of consciousness or irritability<br>GCS 9-13	Awake or can be awakened but fussy<br>GCS 14	Awake or can be awakened and alert<br>GCS 15
E	Temp < 35	Temp > 41<br>In children < 3 mos.:<br>temp < 36 or ≥ 38.5	Children 3 mos. – 3 years:<br>temp ≥ 38.5 and fussy	

* central capillary refill time, if peripheral not ≤ 2 sec

Respiratory rate

Child age	1	2	3	4	3	2	1
0-2 mo.	<10	10-19	20-29	30-60	61-80	81-90	>90
3-11 mo.	<10	10-19	20-29	30-60	61-70	71-80	>80
1-2 years	<10	10-14	15-19	20-30	31-40	41-50	>50
3-7 years	<10	10-12	13-15	16-24	25-30	31-35	>35
≥ 8 years	<8	8-9	10-11	12-25	26-30	31-35	>35

Heart rate

Child age	1	2	3	4	3	2	1
0-2 mo.	<50	50-69	70-89	90-180	181-205	206-230	>230
3-11 mo.	<40	40-59	60-79	80-160	161-190	191-230	>230
1-2 years	<40	40-58	59-74	75-130	131-165	166-200	>200
3-7 years	<40	40-54	55-69	70-110	111-125	126-165	>165
≥ 8 years	<40	40-44	45-49	50-110	111-120	121-140	>140
