# Supplementary material for: Prehospital vital sign monitoring in paediatric patients: an interregional study of educational interventions
Source: Scand J Trauma Resusc Emerg Med. 2023 Jan 14;31:4. doi: 10.1186/s13049-023-01067-z (PMC9839956; doi:10.1186/s13049-023-01067-z)
Supplement: Supplementary file 2 — Additional file 2: Differences between triage scores calculated from the first and last set of vital signs in the intervention region (North Denmark Region). [file 13049_2023_1067_MOESM2_ESM.docx]

**Additional file 2.** Differences between triage scores calculated from the first and last set of vital signs in the intervention region (North Denmark Region).

| **Table A1.** Differences between first and last triage scores^a^ in the intervention region during the pre- and post-intervention periods (n = 7,551) | | | | |
| --- | --- | --- | --- | --- |
|  | Pre-intervention | | Post-intervention | |
|  | (n = 3,841) | | (n = 3,710) | |
| **Incomplete data** (only one set of vital signs) | | | | |
| n (%) | 228 | (5.9) | 140 | (3.8) |
| [95% CI] |  | [5.2;6.7] |  | [3.2;4.4] |
| **Improvement** |  |  |  |  |
| n (%) | 957 | (24.9) | 1,087 | (29.3) |
| [95% CI] |  | [23.6;26.3] |  | [27.9;30.8] |
| **Deterioration** |  |  |  |  |
| n (%) | 330 | (8.6) | 344 | (9.3) |
| [95% CI] |  | [7.7;9.5] |  | [8.4;10.2] |
| **No change** |  |  |  |  |
| n (%) | 1,983 | (51.6) | 1,793 | (48.3) |
| [95% CI] |  | [50.0;53.2] |  | [46.7;49.9] |
| **Missing data** (no vital signs) | |  |  |  |
|  | 343 | (8.9) | 346 | (9.3) |
|  |  | [8.1;9.9] |  | [8.4;10.3] |
| ^a^Calculated from vital signs according to the Danish Regions’ Paediatric Triage Model. | | | | |
